# Lipid-Lowering Efficacy of the Capsaicin in Patients With Metabolic Syndrome: A Systematic Review and Meta-Analysis of Randomized Controlled Trials

**DOI:** 10.3389/fnut.2022.812294

**Published:** 2022-03-01

**Authors:** Zhonghui Jiang, Hua Qu, Gongyu Lin, Dazhuo Shi, Keji Chen, Zhuye Gao

**Affiliations:** ^1^Department of Cardiology, China Academy of Chinese Medical Sciences, Xiyuan Hospital, Beijing, China; ^2^National Clinical Research Center for Chinese Medicine Cardiology, Beijing, China; ^3^Department of Graduate School, Beijing University of Chinese Medicine, Beijing, China

**Keywords:** capsaicin, lipid levels, metabolic syndrome, randomized controlled trials, meta-analysis

## Abstract

**Background:**

Patients with metabolic syndrome (MetS) have increased cardiovascular risk. Capsaicin (CAP) has been shown to reduce lipids, but efficacy for patients with MetS is unknown.

**Methods:**

A systematic review was performed according to PRISMA guidelines, to compare the effects of CAP against a placebo. Differences in the weight mean difference (WMD) with 95% confidence intervals (95% CI) were then pooled using a random effects model.

**Results:**

Nine randomized controlled trials including 461 patients were identified in the overall analysis. CAP significantly decreased total cholesterol (TC) (WMD = −0.48, 95% CI: −0.63 to −0.34, *I*^2^= 0.00%) and low-density lipoprotein cholesterol (LDL-C) (WMD = −0.23, 95% CI: −0.45 to −0.02, *I*^2^ = 68.27%) among patients with MetS. No significant effects of CAP were found on triglycerides (TG) or high-density lipoprotein cholesterol (HDL-C) (WMD = −0.40, 95% CI: −1.50 to 0.71, *I*^2^ = 98.32%; WMD = −0.08, 95% CI: −0.21 to 0.04, *I*^2^ = 86.06%). Subgroup analyses indicated that sex and intervention period were sources of heterogeneity. The results revealed that CAP decreased TG levels in women (WMD = −0.59, 95% CI: −1.07 to −0.10) and intervention period <12 weeks (WMD = −0.65; 95% CI: −1.10 to −0.20). And there was no potential publication bias according to funnel plot, Begg' test and Egger regression test.

**Conclusions:**

CAP supplementation is a promising approach to decreasing TC and LCL-C levels in patients with MetS. However, short-term (<12 weeks) use of CAP in women may also reduce TG levels.

**Systematic Review Registration:**

Identifier: CRD42021228032.

## Introduction

Metabolic syndrome (MetS) represents a cluster of metabolic risk factors, including dyslipidemia (raised triglycerides and lowered high-density lipoprotein cholesterol), obesity (especially abdominal obesity), elevated blood pressure, and dysglycemia (1). It is estimated that patients with MetS are twice as likely to develop cardiovascular disease (CVD) in the next 5 to 10 years compared to those without the syndrome, posing a huge burden on global health and the economy (1). The management of each MetS component has been proved to be effective in reducing the incidence of CVD and reducing the risk for major adverse cardiovascular events (MACE) (2).

Dyslipidemia is an important component of the contemporary consensus definition of MetS and also a major risk factor for CVD, which is a leading cause of death worldwide (3–7). Epidemiological data revealed that in addition to decreased levels of high-density lipoprotein cholesterol (HDL-C), elevated levels of low-density lipoprotein cholesterol (LDL-C), triglycerides (TG) and total cholesterol (TC) are also independent predictors of the risk of CVD (8–12). Therefore, lowering blood lipid levels is of great significance in the management of MetS.

Recently there has been rising attention to single dietary components or natural compounds due to the fact they are inexpensive, readily available, and have beneficial effects in the treatment of various diseases such as MetS. For example, studies have shown that daily intake of red yeast rice can reduce LDL-C levels from 15% to 25% (13). In addition, glucomannan (14, 15), probiotics (14, 16), garlic (17), berberine (18), omega-3 (ω′-3) and fatty acids (19), also have a positive effect on improving MetS.

Capsaicin (CAP) (trans-8-methyl-N-vanillyl-6-nonenamide) is the main component in red chili peppers that give chili peppers their spice, which belongs to the Solanaceae family (20, 21). Previous studies have demonstrated that CAP has antioxidant activity (22), analgesic activity (23, 24), and can aid in lowering rates of obesity (25). The lipid-lowering effects of the CAP remain controversial. Numerous studies have shown that CAP can reduce TC, TG, LDL-C and increase HDL-C levels (20, 26, 27). However, a study by Urbina et al. (28) showed that CAP supplementation does not affect serum TG, TC, LDL-C levels, but does decrease HDL-C levels in serum, which is not consistent with evidence reported in previous studies. There have been no systematic reviews or meta-analyses to summarize the available data. Therefore, we carry out a systematic review and meta-analysis to assess the effects of CAP on lipids in patients with MetS.

## Methods

### Data Sources and Searches

The meta-analysis protocol was registered on PROSPERO (CRD42021228032, https://www.crd.york.ac.uk/prospero/), and conducted following the Preferred Reporting Items for Systematic Reviews and Meta-Analyses (PRISMA) guidelines (29). PubMed, EMBASE, MEDLINE, and the Cochrane Library were systematically searched from inceptions to February 1, 2021. MeSH terms and free words were used reasonably through the characteristics of literature databases. Detailed search strategies are listed in Supplementary Material 1.

### Selection Criteria

The selected studies were screened using the PICOS (participants, interventions, comparisons, outcomes, and study design) criteria:

1. Population: patients with MetS were diagnosed according to recognized diagnostic criteria (IFD, WHO, or NCEP-ATP III) (1).
2. Intervention: circulating CAP supplements, dietary CAP or CAP-related supplements.
3. Comparison: no use of CAP or CAP-related supplements categories of exposure.
4. Outcome: lipid parameters (TC, TG, HDL-C and LDL-C).
5. Study design: randomized controlled trials (RCTs).

### Data Extraction

Two reviewers independently extracted data using standardized data extraction forms. Any disagreements would be resolved by consensus or consulting the third reviewer. If the information is incomplete or unclear, when necessary, the author was contacted. Data extraction included study design type and participant characteristics (age, sex, and country), intervention and placebo details (sample size, study duration, CAP dose, and controls group used). Outcomes included lipid levels (TC, TG, HDL-C, LDL-C).

### Data Synthesis and Analysis

Data for the effect of continuous outcomes were extracted as the weight mean difference (WMD), which represents the mean difference between the intervention and control groups in standard deviation units, with 95% confidence intervals (CIs). Clinical heterogeneity was assessed by *I*^2^ and Chi-squared (χ^2^) test at α = 0.1. When Cochrane's test showed that *I*^2^ <50%, there was no statistical heterogeneity among the studies, and therefore a fixed-effect model (with inverse variance method) was used for meta-analysis. If *I*^2^ ≥ 50%, there was statistical heterogeneity among the studies, and as such the random effects model (DerSimonian and Laird method) was used to analyze the causes of the heterogeneity. Sensitivity analysis was performed by using the leave-one-out method and/ or a subgroup analysis according to that factor. Publication bias was examined using funnel plots, the Begg' test and the Egger regression test. Statistical analysis was conducted using Review Manager, Version 5.3 (Cochrane Collaboration, Oxford, UK) and Stata version 16.0 (Stata Corp., College Station, TX). The protocol for the present meta-analysis was registered on the international prospective register of systematic reviews (PROSPERO, CRD42021228032).

### Quality Assessment

The recommendations of the Cochrane Intervention Systems Review Manual (updated September 2009) were used to evaluate the risk of bias. Studies were stratified as high risk, low risk, or unclear risk. The risk of bias included the following six evaluation criteria: generation of a random sequence, allocation concealment, use of blind method, integrity of result data, and selection of reporting outcome.

## Results

### Data Sources and Search Results

**Figure 1:**
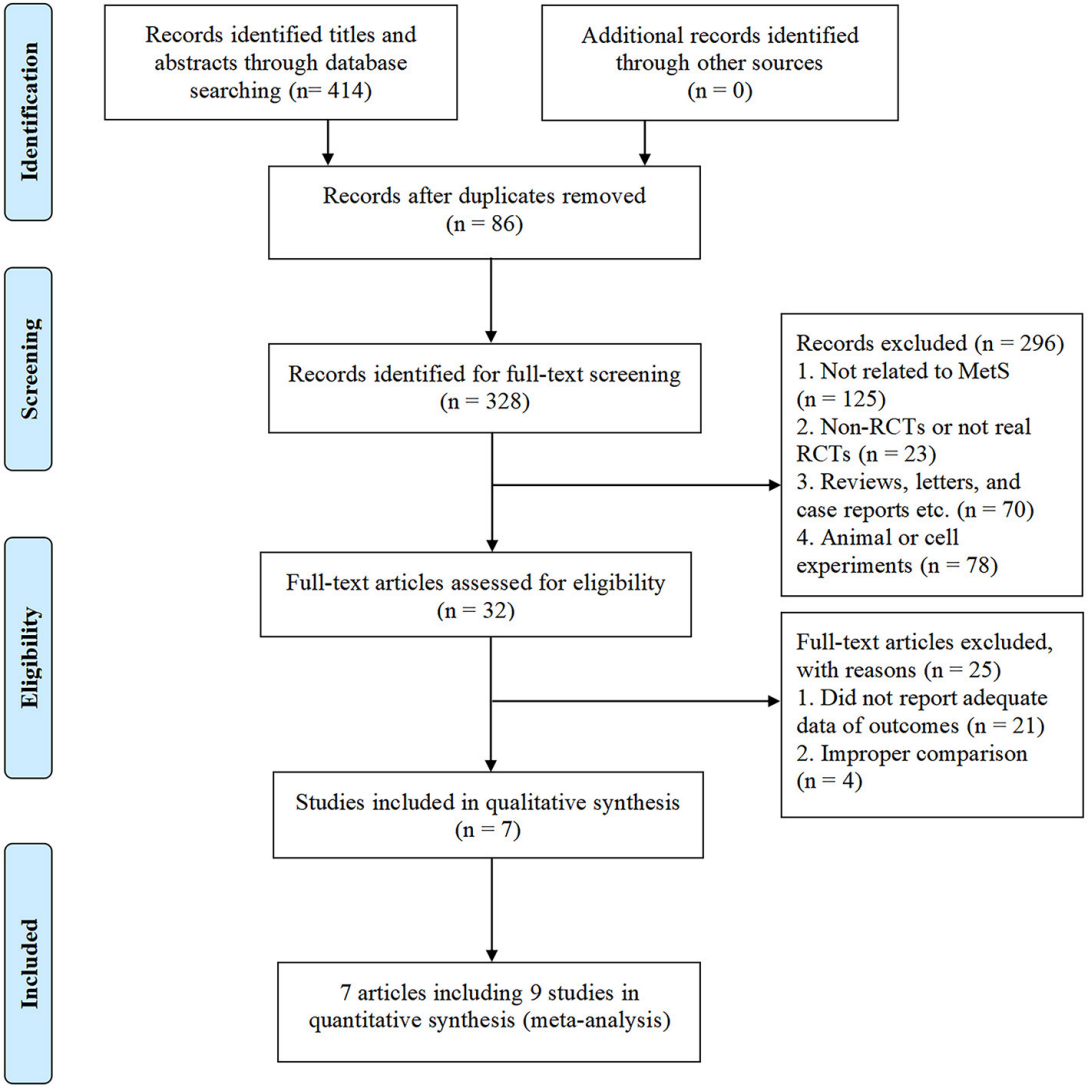
PRISMA 2009 flow diagram for study selection process.

As shown in Figure 1, a total of 414 potentially relevant studies were identified in our initial literature search. We evaluated 328 potentially related articles for eligibility after removing the duplicates of 86 studies from different databases. After screening the titles and abstracts of these studies, we excluded 296 studies for the following reasons: subject was not related to MetS (*n* = 125); study not a RCTs (*n* = 23); reviews, letters, and case reports etc. (*n* = 70); animal or cell experiments (n = 78). Of the retrieved studies, a total of 32 met our inclusion criteria. However, 25 studies were excluded because they did not have sufficient data of outcomes (n = 21) or improper comparison (*n* = 4). Finally, seven studies entered into our meta-analysis, involving 461 patients (227 [49.2%] in the CAP group, 234 [50.8%] in the control group). Three studies (28, 30, 31) were conducted and published in full in the United States, one in Korea (32), one in the Netherlands (33), one in Iran (34), and one in China (35). The average sample size of the trials was 51 participants (ranging from 36 to 87 participants per trial). The course of treatment fluctuated between 4-weeks and 13-weeks. In one of the included trials (28), two different doses of CAP (2 and 4 mg) were administered, hence we considered it as two separate studies. In another trial (30), results were divided by sex despite the same intervention, so we also treated this study as two separate studies. The characteristics of the included trials are shown in Table 1.

**Table 1:** Characteristics of included capsaicin RCTs for MetS.

**References**	**Number of** **participants (T/C)**	**Population**	**Age(years)** **(T/C)**	**Intervention group (T/C)**	**Duration** **(weeks)**	**Main outcomes**
Arent et al. (30) (United States)	18/18	Overweight men	18–50	METABO (4 capsules/d)/Placebo	8	TC, LDL-C, HDL-C, TG
Arent et al. (30) (United States)	18/18	Overweight women	18–50	METABO (4 capsules/d)/Placebo	8	TC, LDL-C, HDL-C, TG
Cha et al. (32) (Korea)	30/30	Overweight subjects	19–65	KCJ (3,200 mg/d)/Placebo	12	TC, LDL-C, HDL-C, TG
Lejeune et al. (33) (The Netherlands)	40/47	Overweight subjects	18–60	Capsaicin (135 mg/d)/Placebo	13	TG
Lopez et al. (31) (United States)	27/18	Overweight subjects	21–45	METABO (4 capsules/d)/Placebo	8	TC, LDL-C, HDL-C, TG
Taghizadeh et al. (34) (Iran)	25/25	Overweight women	18–50	Capsaicin (25 mg/d)/Placebo	8	TC, LDL-C, HDL-C, TG
Urbina et al. (28) (United States)	27/28	Overweight subjects	18–56	Capsaicinoid (2 mg/d)/Placebo	12	LDL-C, HDL-C, TG
Urbina et al. (28) (United States)	22/28	Overweight subjects	18–56	Capsaicinoid (4 mg/d)/Placebo	12	LDL-C, HDL-C, TG
Yuan et al. (35) (China)	20/22	Women with gestational diabetes mellitus	(31.1 ± 4.4)/ (29.8 ± 4.5)	Capsaicin (5 mg/d)/Placebo	4	TC, LDL-C, HDL-C, TG

*RCTs, randomized controlled trials; MetS, metabolic syndrome; T/C, treatment/control; METABO, consisted of raspberry ketone, caffeine, capsaicin, garlic organosulfur compounds, gingerols, shogaols, Citrus aurantium and related alkaloids, B vitamins, and chromium; KCJ, a fermented soybean-based red pepper paste, containing powdered red pepper 11.9g; TC, total cholesterol; LDL-C, low-density lipoprotein-cholesterol; HDL-C, high-density lipoprotein-cholesterol; TG, triglycerides*.

### Quality of Included Studies

**Figure 2:**
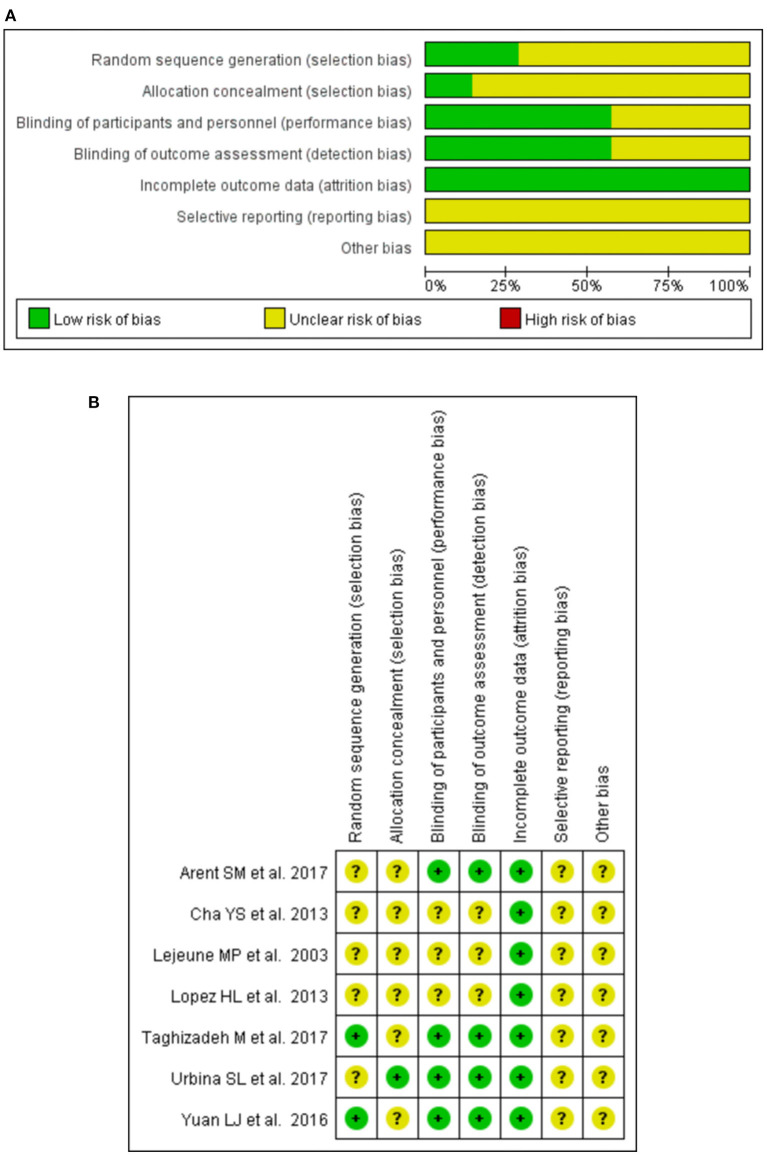
Graph of bias risk assessment for included studies by the Cochrane Collaboration's tool. **(A)** Risk of bias graph. **(B)** Risk of bias summary.

The assessment of the risk of bias for all included trials is shown in Figures 2A,B. All of the included studies reported randomly assigned participants, two (34, 35) of them described methodological operations for random sequence generation (using a computer-generated list of random numbers), and the remaining studies mentioned they were “random,” did not report it in detail. Two studies (28) reported the method of allocation concealment (by using numbered bottles). All trials were double-blind, of these, six studies (28, 30, 34, 35) described the specific blinding of participants and personnel, and detailed the blinding of the outcome assessment. Clinical trial registration and pre-published protocol were reported by the authors of eight studies (28, 30–32, 34, 35), but it was not feasible to effectively assess whether there was a risk of selective reporting bias.

### Effects of CAP on Lipid Levels

#### Total Cholesterol

**Figure 3:**
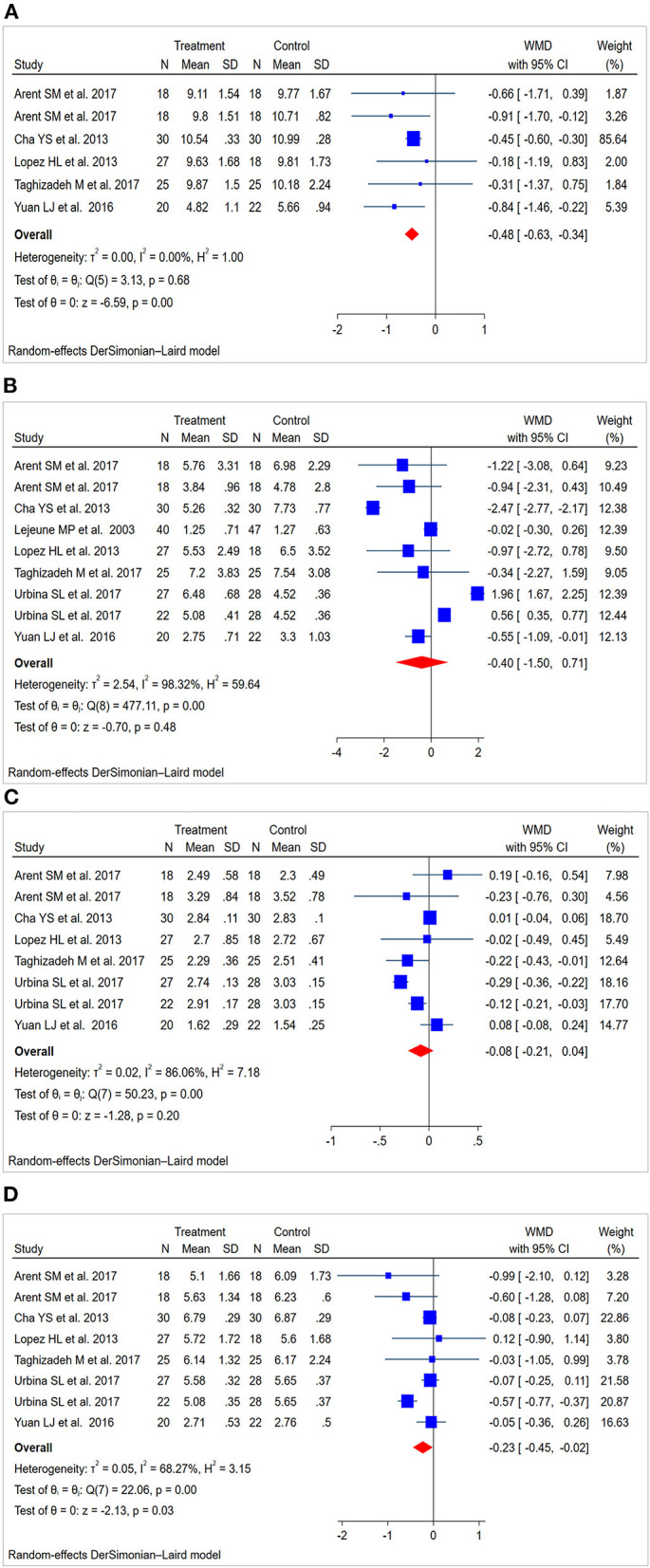
Forest plot for lipid levels: capsaicin vs. placebo (random-effect model). **(A)** Funnel plot for total cholesterol (TC). **(B)** Funnel plot for triglycerides (TG). **(C)** Funnel plot for high-density lipoprotein cholesterol (HDL-C). **(D)** Funnel plot for low-density lipoprotein cholesterol (LDL-C).

**Figure 4:**
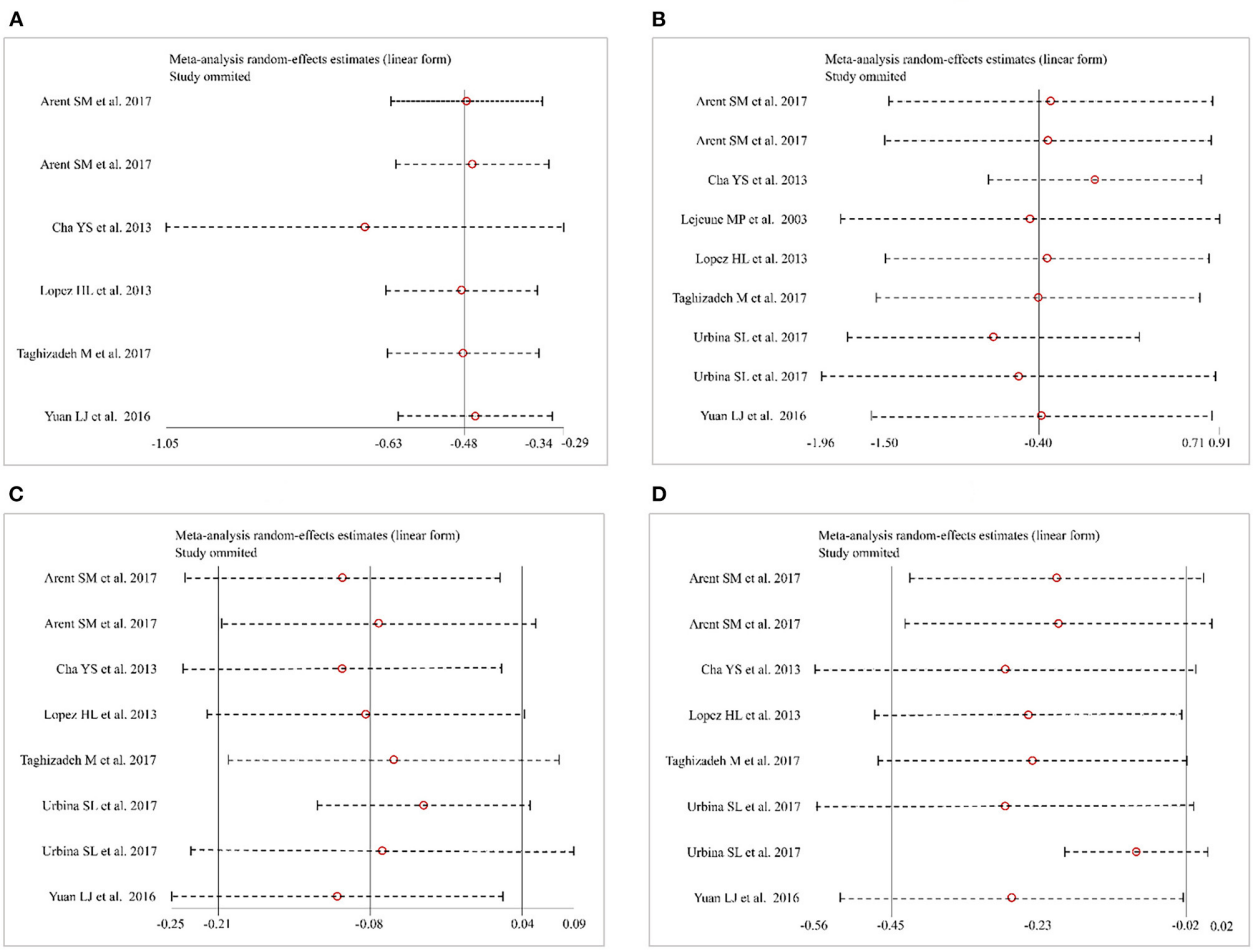
Sensitivity analysis. **(A)** Total cholesterol (TC). **(B)** Triglycerides (TG). **(C)** High-density lipoprotein cholesterol (HDL-C). **(D)** Low-density lipoprotein cholesterol (LDL-C).

**Figure 5:**
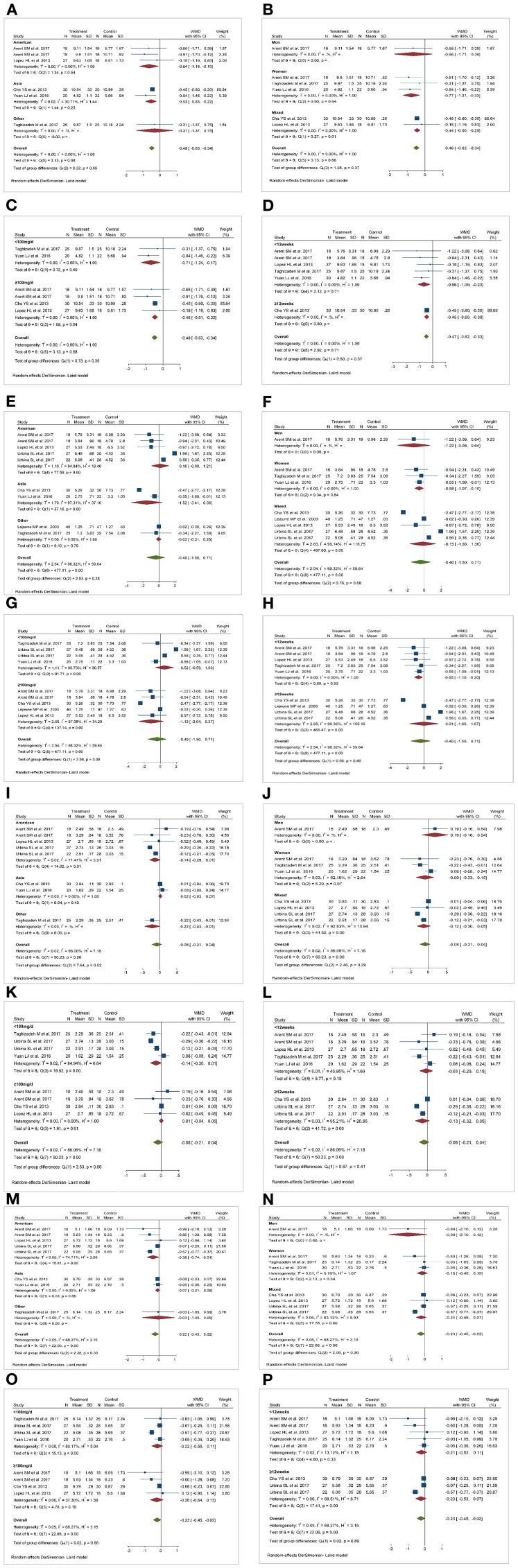
Results of subgroup analysis. **(A,E,I,M)** Effect of capsaicin (CAP) on total cholesterol (TC), triglycerides (TG), high-density lipoprotein cholesterol (HDL-C), and low density lipoprotein cholesterol (LDL-C) in different races. **(B,F,J,N)** Effect of CAP on TC, TG, HDL-C and LDL-C in different genders. **(C,G,K,O)** Effect of dose of CAP on TC, TG, HDL-C and LDL-C. **(D,H,L,P)** Effect of duration of CAP intervention on TC, TG, HDL-C and LDL-C. WMD, weighted mean difference.

Six studies (30–32, 34, 35) (138 patients in the CAP supplementation group vs. 131 patients in the placebo group) evaluated the effects of CAP supplementation on TC levels among patients with MetS. The overall results of the random-effect model exhibited a favorable effect on reducing TC levels following CAP supplementation (WMD = −0.48; 95% CI: −0.63 to −0.34; *P* = 0.00; *I*^2^ = 0.00%) (Figure 3A). The results of leave-one-out sensitivity analysis support the robustness of our findings (Figure 4A). Inter-group heterogeneity changed after subgroup analysis based on race, sex, dose and duration of CAP supplementation, but there was no significant difference in TC levels before and after the subgroup analysis (Figure 5).

#### Triglycerides

Nine studies (28, 30–35) (227 patients in the CAP supplementation group vs. 234 patients in the placebo group) compared the effects of CAP and placebo on TG levels in patients with MetS. The results showed that CAP had no significant effect on TG levels compared with placebo (WMD = −0.40; 95% CI: −1.50 to 0.71; *P* = 0.48; *I*^2^ = 98.32%) (Figure 3B). Sensitivity analysis showed that the results did not change before and after sensitivity analysis (Figure 4B).

Figure 5 summarizes the subgroup analysis results of the effects of CAP on TG levels in patients with MetS. TG levels in women were significantly decreased after CAP supplementation (WMD = −0.59; 95% CI: −1.07 to −0.10). Furthermore, serum TG levels decreased after CAP supplementation for <12 weeks (WMD = −0.65; 95% CI: −1.10 to −0.20).

#### High-Density Lipoprotein Cholesterol

Eight studies (28, 30–32, 34, 35) (187 patients in the CAP supplementation group vs. 187 patients in the placebo group) reported the effects of CAP on serum HDL-C. The random effects model showed that the pooled mean effect size was not significant (WMD = −0.08; 95% CI: −0.21 to 0.04; *P* = 0.20), with significant heterogeneity (*I*^2^= 86.06%) (Figure 3C). The results of the sensitivity analysis were not altered after excluding the individual trials (Figure 4C). The heterogeneity changed with differences in race, gender, intervention time groups and dose, but there were no significant differences before and after subgroup analysis (Figure 5).

#### Low-Density Lipoprotein Cholesterol

Eight studies (28, 30–32, 34, 35) indicated beneficial results from taking CAP supplementation (*n* = 187), as seen by a reduction in serum LDL-C levels, compared to that with placebo group (*n* = 187) (WMD = −0.23; 95% CI: −0.45 to −0.02, *P* = 0.03; *I*^2^ = 68.27%) using the random-effect model (Figure 3D). Sensitivity analysis showed no significant change in the overall estimate of effect size after the elimination of individual trials (Figure 4D). The subgroup analysis of the LDL-C levels showed no significant differences within subgroups based on the dose of CAP, duration of CAP use, race or gender (Figure 5).

### Publication Bias

**Figure 6:**
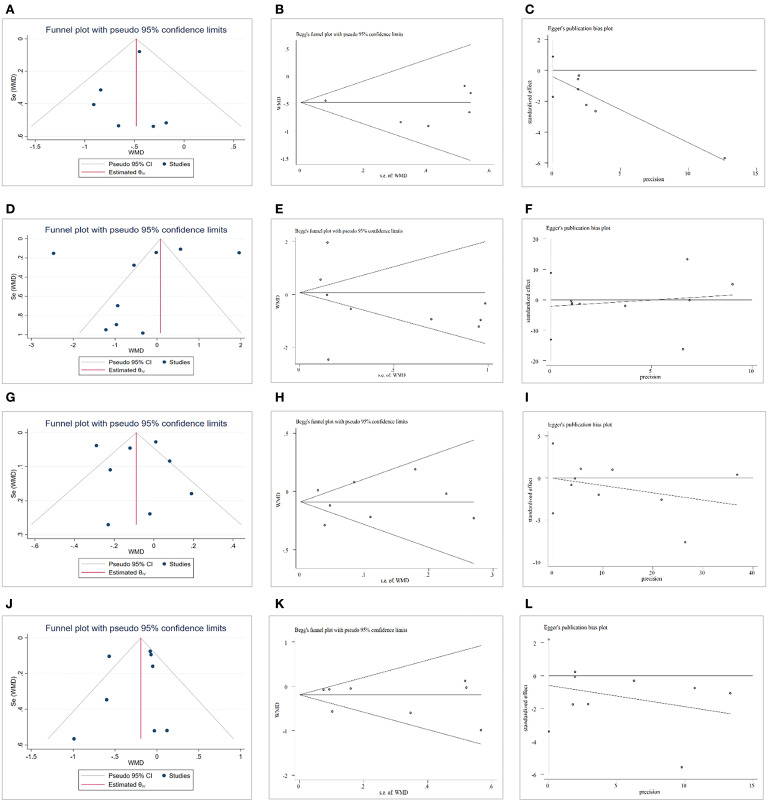
Publication bias. **(A)** Funnel plot for total cholesterol (TC). **(B)** Begg' test for TC. **(C)** Egger test for TC. **(D)** Funnel plot for triglycerides (TG). **(E)** Begg' test for TG. **(F)** Egger test for TG. **(G)** Funnel plot for high-density lipoprotein cholesterol (HDL-C). **(H)** Begg' test for HDL-C. **(I)** Egger test for HDL-C. **(J)** Funnel plot for low-density lipoprotein cholesterol (LDL-C). **(K)** Begg' test for LDL-C. **(L)** Egger test for LDL-C.

A funnel plot, Begg' test and Egger regression test were used to evaluate the effects of CAP on TC levels, and no publication bias was found (Egger regression test, coefficient, −0.42; 95% CI, −1.72 to 0.89; *P* = 0.43). For the effects of CAP on TG and HDL-C levels, there was no evidence of publication bias according to the results of a funnel plot, Begg' test and Egger regression test (coefficient, −2.15; 95% CI, −13.08 to 8.79; *P* = 0.66; coefficient, −0.07; 95% CI, −4.22 to 4.08; *P* = 0.97). Lastly, no potential publication bias for LDL-C was identified according to funnel plot, Begg' test and Egger regression test (coefficient, −0.59; 95% CI, −3.39 to 2.21; *P* = 0.62) (Figure 6).

## Discussion

To the best of our knowledge, this is the first systematic review and meta-analyses of RCTs evaluating the effects of CAP supplementation on lipid levels among patients with MetS. A total of nine studies (involving 461 patients) were included in this meta-analysis. The main finding of our meta-analysis was that CAP supplementation may have beneficial therapeutic effects in reducing TC and LDL-C levels. No significant effects of CAP were found regarding TG and HDL-C levels. However, subgroup analyses revealed that CAP reduced TG levels in women and at <12 weeks of the intervention.

Many small studies have reported that CAP might decrease lipid levels among patients with MetS. This effect has also been proposed CAP as one of the agents treating dyslipidemia. In most instances, however, the studies regarding CAP and lipid levels had methodological limitations (mainly owing to small numbers of patients included), leaving this hypothesis unproven. Our meta-analysis pooled all the RCTs regarding the effects of CAP on lipid levels, and the results showed that CAP supplementation may be beneficial in reducing TC, and LDL-C. Overall, CAP may be a complementary approach in the patients with dyslipidemia who cannot be treated with statins or other LDL-C-lowering therapies.

It is essential to actively manage risk factors for MetS, such as dyslipidemia. Studies have shown that lowering atherogenic cholesterol levels can effectively reduce morbidity and mortality of CVD (36–38). The ATP-III guidelines emphasized that LDL-C reduction is the primary target of lipid management in MetS, and low HDL-C and TG are secondary targets (39). Large LDL-C reductions, such as 2 to 3 mmol/L (77.4–116.1 mg/dL), can reduce the relative risk of CVD by 40–50% (37). The availability and use of lipid-lowering medication, such as statin therapy and ezetimibe, or proprotein convertase subtilisin-kexin type 9 (PCSK9) inhibitors, significantly reduces lipid levels. In turn, reducing the number of patients with hyperlipidemia and therefore the risk of an acute cardiovascular event (40, 41). Statin therapies also generally reduced the risk of an acute cardiovascular event by 25 to 45%, which was noted over 5 years of follow-ups (6). However, despite the existence of effective treatments and well-established treatment guidelines, lipid abnormalities are still very common in adults, with an estimated 53% (105.3 M) of U.S. adults having at least one lipid abnormality, 27% (53.5 million) having high LDL-C, 23% (46.4 million) having low HDL, and 30% (58.9 million) with high TG (42). A clinical guideline for the management of dyslipidemia conducted by Downs and O'Malley showed that 10 to 20% of patients using statins experienced muscle-related symptoms (43). Hereby, our meta-analysis showed that CAP can improve dyslipidemia and has the advantages of a lower price and easy availability. For these reasons, CAP supplementation as an adjunct nutritional therapy for the treatment of dyslipidemia in MetS patients is easy to implement and may lead to better compliance in patients with MetS.

There are several mechanisms which could potentially explain the effects of CAP on lipid levels. It has been shown that CAP plays a role in countering the detrimental effects of a high-fat diet, such as glucose intolerance and/or hypercholesterolemia. It does this primarily by increasing the expression of metabolically important thermogenic genes, including uncoupling protein 1 (UCP-1), bone morphogenetic protein 8b (BMP 8b), Sirtuin1 (SIRT-1), peroxisome proliferator-activated receptor-γ co-activator-1α (PGC-1α), and positively regulated domain containing zinc finger protein 16 (PRDM-16) (44). CAP activates its receptor transient receptor vanilloid subtype 1 (TRPV1), which can activate sympathetically-mediated brown adipose tissue (BAT) thermogenesis and reduce body fat (45). In addition, CAP inhibits the expression of peroxisome proliferator-activated receptor-γ (PPARγ), CCAAT-enhancer-binding protein-α (C/EBP-α) and leptin; but induces up-regulation of adiponectin at the protein level. Therefore, it can effectively induce apoptosis of 3T3-L1 pre-adipocytes and adipocytes; and inhibit adipogenesis *in vitro* (46).

The meta-analysis has a number of limitations, of which heterogeneity across the included studies is the most important. We conducted a sensitivity and subgroup analysis to determine the factors (race, gender, dose, and duration, etc.) that might cause large heterogeneity and thus to explore the source of heterogeneity. Heterogeneity changed after analysis, but the overall results were stable and reliable. In clinical practice, disparate formulations and delivery routes of CAP may affect the results, which should be noted in future research. Second, this meta-analysis was limited by the small number of studies and the small size of existing RCTs. Therefore, additional studies are needed to confirm our findings and to expand our understanding of CAP.

In conclusion, the findings of this meta-analysis demonstrated that CAP supplementation is effective in improving lipid levels and should be considered in the prevention and treatment of MetS. Large-scale, high-quality, and precise RCTs are needed to further demonstrate the effects of CAP on lipid levels, and we will follow up on this study.

## Data Availability Statement

The original contributions presented in the study are included in the article/Supplementary Materials, further inquiries can be directed to the corresponding author/s.

## Author Contributions

ZJ and HQ were involved in the conception and design. ZJ, HQ, GL, and ZG were involved in literature retrieval, data collection, extraction and analysis. KC and DS were involved in systematic review and meta-analysis. KC and ZG are responsible for the final approval of the version to be published. All authors revised and approved the final version of the manuscript.

## Funding

This work was financially supported by the National Science and Technology Major Project of the Ministry of Science and Technology of China (2019ZX09201005-002-006), Beijing Traditional Chinese Medicine Science and Technology Development Fund Project (JJ-2020-79), and the Capital Health Research and Development of Special (2018–1–4171).

## Conflict of Interest

The authors declare that the research was conducted in the absence of any commercial or financial relationships that could be construed as a potential conflict of interest.

## Publisher's Note

All claims expressed in this article are solely those of the authors and do not necessarily represent those of their affiliated organizations, or those of the publisher, the editors and the reviewers. Any product that may be evaluated in this article, or claim that may be made by its manufacturer, is not guaranteed or endorsed by the publisher.
