# Supplementary material for: Lipid-Lowering Efficacy of the Capsaicin in Patients With Metabolic Syndrome: A Systematic Review and Meta-Analysis of Randomized Controlled Trials
Source: Front Nutr. 2022 Mar 1;9:812294. doi: 10.3389/fnut.2022.812294 (PMC8923259; doi:10.3389/fnut.2022.812294)
Supplement: Supplementary file 1 [file Data_Sheet_1.docx]

**Supplementary Material 1. Search strategies for the English language databases**

| Database | Search | Search Strings |
| --- | --- | --- |
| PubMed | #1 | ((((((((((((((((Capsaicine) OR 8-Methyl-N-Vanillyl-6-Nonenamide) OR 8 Methyl N Vanillyl 6 Nonenamide) OR Antiphlogistine Rub A-535 Capsaicin) OR Axsain) OR Zacin) OR Capsidol) OR Zostrix) OR Capzasin) OR Gelcen) OR Katrum) OR NGX-4010) OR NGX 4010) OR NGX4010) OR Capsicum Farmaya) OR Capsin) OR Capsaicin [All Fields] |
|  | #2 | ((((((((Total cholesterol) OR Epicholesterol) OR Cholesterin) OR Cholestenone) OR Cholesteryl ester transfer protein) OR Cholesteryl ester) OR Cholesterol ester) OR cholesterol) OR TC [All Fields] |
|  | #3 | ((((Triglycerides) OR Triacylglycerol) OR Triacylglycerols) OR Triglyceride) OR TG [All Fields] |
|  | #4 | ((((((((((((((((((High-density lipoprotein cholesterol) OR Lipoproteins, HDL) OR HDL Lipoproteins) OR High-Density Lipoprotein) OR Lipoprotein, High-Density) OR High-Density Lipoproteins) OR High Density Lipoproteins) OR Lipoproteins, High-Density) OR alpha-Lipoproteins) OR alpha Lipoproteins) OR Heavy Lipoproteins) OR Lipoproteins, Heavy) OR High Density Lipoprotein) OR Density Lipoprotein, High) OR Lipoprotein, High Density) OR alpha-Lipoprotein) OR alpha Lipoprotein) OR alpha-1 Lipoprotein) OR HDL-C [All Fields] |
|  | #5 | (((((((((Low-density lipoprotein cholesterol) OR Cholesterol, LDL) OR Low Density Lipoprotein Cholesterol) OR beta-Lipoprotein Cholesterol) OR Cholesterol, beta-Lipoprotein) OR beta Lipoprotein Cholesterol) OR LDL Cholesterol) OR Cholesteryl Linoleate, LDL) OR LDL Cholesteryl Linoleate) OR LDL-C [All Fields] |
|  | #6 | ((((((((((((((((((((((((((((((((((((((((((((((((((((((((((((((((((((((((((((((((((((((((Syndrome, Metabolic) OR Syndromes, Metabolic) OR Metabolic Syndrome X) OR Insulin Resistance Syndrome X) OR Syndrome X, Metabolic) OR Syndrome X, Insulin Resistance) OR Metabolic X Syndrome) OR Syndrome, Metabolic X) OR X Syndrome, Metabolic) OR Dysmetabolic Syndrome X) OR Syndrome X, Dysmetabolic) OR Reaven Syndrome X) OR Syndrome X, Reaven) OR Metabolic Cardiovascular Syndrome) OR Cardiovascular Syndrome, Metabolic) OR Cardiovascular Syndromes, Metabolic) OR Syndrome, Metabolic Cardiovascular) OR Cardiometabolic Syndrome) OR Cardiometabolic Syndromes) OR Syndrome, Cardiometabolic) OR Syndromes, Cardiometabolic) OR Metabolic Syndromes) Abdominal Obesities) OR Obesities, Abdominal) OR Central Obesity) OR Central Obesities) OR Obesities, Central) OR Obesity, Central) OR Obesity, Visceral) OR Visceral Obesity) OR Obesities, Visceral) OR Visceral Obesities) OR Abdominal Obesity) OR Dyslipidemias) OR Dyslipoproteinemias) OR Dyslipoproteinemia) OR Dyslipidemia) OR Blood Pressure, High) OR Blood Pressures, High) OR High Blood Pressure) OR High Blood Pressures) OR Hypertension) OR Hyperglycemias) OR Hyperglycemia, Postprandial) OR Hyperglycemias, Postprandial) OR Postprandial Hyperglycemias) OR Postprandial Hyperglycemia) OR Hyperglycemia) OR Resistance, Insulin) OR Insulin Sensitivity) OR Sensitivity, Insulin) OR Insulin Resistance) OR Diabetes Mellitus, Noninsulin-Dependent) OR Diabetes Mellitus, Ketosis-Resistant) OR Diabetes Mellitus, Ketosis Resistant) OR Ketosis-Resistant Diabetes Mellitus) OR Diabetes Mellitus, Non-Insulin Dependent) OR Diabetes Mellitus, Non-Insulin-Dependent) OR Non-Insulin-Dependent Diabetes Mellitus) OR Diabetes Mellitus, Stable) OR Stable Diabetes Mellitus) OR Diabetes Mellitus, Type II) OR NIDDM) OR Diabetes Mellitus, Noninsulin Dependent) OR Diabetes Mellitus, Maturity-Onset) OR Diabetes Mellitus, Maturity Onset) OR Maturity-Onset Diabetes Mellitus) OR Maturity Onset Diabetes Mellitus) OR MODY) OR Diabetes Mellitus, Slow-Onset) OR Diabetes Mellitus, Slow Onset) OR Slow-Onset Diabetes Mellitus) OR Type 2 Diabetes Mellitus) OR Noninsulin-Dependent Diabetes Mellitus) OR Noninsulin Dependent Diabetes Mellitus) OR Maturity-Onset Diabetes) OR Diabetes, Maturity-Onset) OR Maturity Onset Diabetes) OR Type 2 Diabetes) OR Diabetes, Type 2) OR Diabetes Mellitus, Adult-Onset) OR Adult-Onset Diabetes Mellitus) OR Diabetes Mellitus, Adult Onset) OR Diabetes Mellitus, Type 2) OR Cardiovascular Diseases) OR Disease, Cardiovascular) OR Diseases, Cardiovascular) OR Cardiovascular Disease) OR Metabolic Syndrome [All Fields] |
|  | #7 | #2 OR #3 OR #4 OR #5 AND #6 |
|  | #8 | randomized controlled trial[pt] |
|  | #9 | randomized clinical trial[pt] |
|  | #10 | controlled clinical trial[pt] |
|  | #11 | randomized[tiab] |
|  | #12 | placebo[tiab] |
|  | #13 | randomly[tiab] |
|  | #14 | trial[tiab] |
|  | #15 | groups[tiab] |
|  | #16 | #8 OR #9 OR #10 OR #11 OR #12 OR #13 OR #14 OR #15 |
|  | #17 | #1 AND #7 AND #16 |
| EMBASE | #1 | 'Capsaicin'/exp OR Capsaicin |
|  | #2 | 'Capsaicine'/exp OR Capsaicine |
|  | #3 | '8-Methyl-N-Vanillyl-6-Nonenamide'/exp OR 8-Methyl-N-Vanillyl-6-Nonenamide |
|  | #4 | 'Antiphlogistine Rub A-535 Capsaicin'/exp OR Antiphlogistine Rub A-535 Capsaicin |
|  | #5 | 'Axsain'/exp OR Axsain |
|  | #6 | 'Zacin'/exp OR Zacin |
|  | #7 | 'Capsidol'/exp OR Capsidol |
|  | #8 | 'Zostrix'/exp OR Zostrix |
|  | #9 | 'Capzasin'/exp OR Capzasin |
|  | #10 | 'Gelcen'/exp OR Gelcen |
|  | #11 | 'Katrum'/exp OR Katrum |
|  | #12 | 'NGX-4010'/exp OR NGX-4010 |
|  | #13 | 'Capsicum Farmaya'/exp OR Capsicum Farmaya |
|  | #14 | 'Capsin'/exp OR Capsin |
|  | #15 | #1 OR #2 OR #3 OR #4 OR #5 OR #6 OR #7 OR #8 OR #9 OR #10 OR #11 OR #12 OR #13 OR #14 |
|  | #16 | 'Total cholesterol'/exp OR Total cholesterol |
|  | #17 | 'Triglycerides'/exp OR Triglycerides |
|  | #18 | 'High-density lipoprotein cholesterol'/exp OR High-density lipoprotein cholesterol |
|  | #19 | 'Low-density lipoprotein cholesterol'/exp OR Low-density lipoprotein cholesterol |
|  | #20 | #16 OR #17 OR #18 OR #19 |
|  | #21 | 'Metabolic Syndromes'/exp OR Metabolic Syndromes |
|  | #22 | 'Abdominal Obesity'/exp OR Abdominal Obesity |
|  | #23 | 'Dyslipidemia '/exp OR Dyslipidemia |
|  | #24 | 'Hypertension '/exp OR Hypertension |
|  | #25 | 'Hyperglycemia '/exp OR Hyperglycemia |
|  | #26 | 'Insulin Resistance '/exp OR Insulin Resistance |
|  | #27 | 'Diabetes Mellitus, Type 2'/exp OR Diabetes Mellitus, Type 2 |
|  | #28 | 'Cardiovascular Disease'/exp OR Cardiovascular Disease |
|  | #29 | #21 OR #22 OR #23 OR #24 OR #25 OR #26 OR #27 OR #28 |
|  | #30 | 'clinical trial' |
|  | #31 | #15 AND #20 AND #29 AND #30 |
| Cochrane Library | #1 | MeSH descriptor: [Capsaicin] explode all trees |
|  | #2 | MeSH descriptor: [Capsaicine] explode all trees |
|  | #3 | (8-Methyl-N-Vanillyl-6-Nonenamide): ti,ab,kw |
|  | #4 | (Antiphlogistine Rub A-535 Capsaicin): ti,ab,kw |
|  | #5 | (Axsain): ti,ab,kw |
|  | #6 | (Zacin): ti,ab,kw |
|  | #7 | (Capsidol): ti,ab,kw |
|  | #8 | (Zostrix): ti,ab,kw |
|  | #9 | (Capzasin): ti,ab,kw |
|  | #10 | (Gelcen): ti,ab,kw |
|  | #11 | (Katrum): ti,ab,kw |
|  | #12 | (NGX-4010): ti,ab,kw |
|  | #13 | (Capsicum Farmaya): ti,ab,kw |
|  | #14 | (Capsin): ti,ab,kw |
|  | #15 | #1 or #2 or #3 or #4 or #5 or #6 or #7 or #8 or #9 or #10 or #11 or #12 or #13 or #14 |
|  | #16 | MeSH descriptor: [Total cholesterol] explode all trees |
|  | #17 | MeSH descriptor: [Triglycerides] explode all trees |
|  | #18 | MeSH descriptor: [High-density lipoprotein cholesterol] explode all trees |
|  | #19 | MeSH descriptor: [Low-density lipoprotein cholesterol] explode all trees |
|  | #20 | #16 or #17 or #18 or #19 |
|  | #21 | MeSH descriptor: [Metabolic Syndromes] explode all trees |
|  | #22 | MeSH descriptor: [Abdominal Obesity] explode all trees |
|  | #23 | MeSH descriptor: [Dyslipidemia] explode all trees |
|  | #24 | MeSH descriptor: [Hypertension] explode all trees |
|  | #25 | MeSH descriptor: [Hyperglycemia] explode all trees |
|  | #26 | MeSH descriptor: [Insulin Resistance] explode all trees |
|  | #27 | MeSH descriptor: [Diabetes Mellitus, Type 2] explode all trees |
|  | #28 | MeSH descriptor: [Cardiovascular Disease] explode all trees |
|  | #29 | #21 #22 or #23 or #24 or #25 or #26 or #27 or #28 |
|  | #30 | #15 and # 20 and #29 |
